# Staged Endovascular Repair of Aortic Coarctation followed by Double Valve Surgery

**DOI:** 10.1055/s-0040-1721749

**Published:** 2021-03-24

**Authors:** Dimitrios V. Avgerinos, Rajeev Dayal, Charles Mack, Samuel Lang, Konstantinos S. Mylonas

**Affiliations:** 1 Department of Cardiothoracic Surgery, New York Presbyterian Medical Center, Weill Cornell College of Medicine, New York, New York; 2 Department of Vascular Surgery, New York Presbyterian Medical Center, Weill Cornell College of Medicine, New York, New York; 3 Department of Surgery, School of Medicine, National and Kapodistrian University of Athens, Athens, Greece

**Keywords:** aortic coarctation, aortic valve insufficiency, mitral valve insufficiency, TEVAR, surgery

## Abstract

We present a unique case of late diagnosis of coarctation of the aorta in an adult, presenting with congestive heart failure associated with severe aortic and mitral valve insufficiency. To minimize operative risk, staged endovascular repair of the coarctation was initially performed, followed by aortic valve replacement and mitral valve repair. Six months postoperatively, the 41-year-old patient remains completely asymptomatic.

## Introduction

Coarctation of the aorta (CoA) accounts for 5 to 8% of congenital heart defects and is typically diagnosed in infancy or childhood. Although rare reports of acquired CoA exist, the vast majority of cases are congenital. CoA occurs due to failure in development of the fourth and sixth pharyngeal arches. Bicuspid aortic valve is the most common cardiac comorbidity seen in association with aortic coarctation. 1

The clinical presentation of CoA in adulthood varies. The earliest clinical sequela of untreated CoA is secondary hypertension. Neglected cases can be further complicated by congestive heart failure, premature coronary artery disease, stroke, aortic dissection, and sudden death. 2 Unrepaired CoA has a mortality rate approaching 70 to 80% by the fifth decade of life. Open surgical repair remains the gold standard, either in the form of resection and end-to-end anastomosis, interposition graft, or subclavian flap aortoplasty. 3 In the last few years, thoracic endovascular aortic repair (TEVAR) has been successfully reported in carefully selected adult patients with primary or recurrent CoA. 4 5 6

We present a unique case of a late diagnosis of CoA presenting with congestive heart failure associated with severe aortic and mitral valve insufficiency, treated with staged endovascular repair of the CoA followed by aortic valve replacement and mitral valve repair.

## Case Presentation

**Fig. 1:**
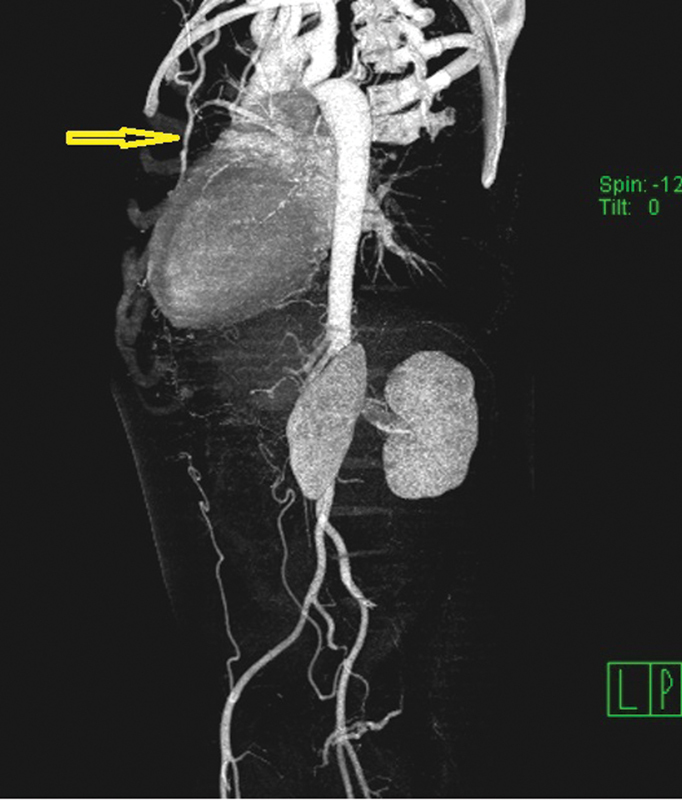
Computed tomography angiogram with three-dimensional reconstruction showing coarctation of the aorta at the site of the isthmus with enlarged left internal mammary artery (arrow).

A 41-year-old male, with past medical history significant only for hypertension (systolic blood pressure, 160–180 mm Hg; diastolic blood pressure, 100–120 mm Hg), presented to the emergency department of our hospital with shortness of breath. Incidentally, for the past few weeks, the patient had been experiencing new-onset dyspnea. Transesophageal echocardiogram revealed a bicuspid aortic valve with severe aortic insufficiency, severe mitral valve regurgitation, and significantly reduced left ventricular ejection fraction (20%). A computed tomographic angiogram revealed aortic coarctation with aortic diameter of 7.7 mm at the site of the isthmus ( Fig. 1 ). There was a 70 mm Hg gradient at the site of the coarctation.

**Fig. 2:**
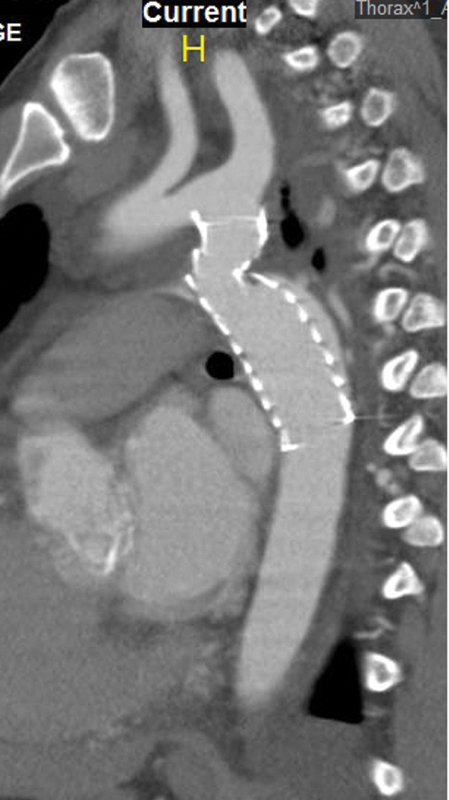
Postdeployment of a 24 mm × 100 mm thoracic aortic graft (Gore, Newark, DE).

It was decided that the patient would benefit from a staged approach, with the coarctation repaired first, followed by aortic valve surgery a few weeks later. Consideration was given to simultaneous valve surgery and frozen elephant trunk; however, it was thought that the significant increase of the cardiopulmonary bypass time would add considerable morbidity. After obtaining informed consent, the patient underwent successful endovascular repair of the aortic coarctation with balloon dilatation of the strictured aorta using a 12 mm × 40 mm standard angioplasty balloon (Boston Scientific, Marlborough, MA), followed by deployment of a 24 mm × 100 mm covered thoracic aortic graft (Gore, Newark, DE), followed by trilobe balloon catheter (Gore, Newark, DE; Fig. 2 ). The predilation was done to ensure that the stent graft could be delivered through the stenotic area as complete as possible expansion of the graft. A 24 mm × 100 mm TEVAR graft was chosen as the proximal aortic diameter was 17 mm and the distal diameter was 28 mm. As the purpose of the procedure was elimination of the coarctation, ensuring a proximal seal and elimination of the gradient was paramount, and allowing the graft to be unopposed in the aorta distal to the coarctation was acceptable. Extending the graft distally beyond the area of dilatation could be performed to eliminate the risk of type IB endoleak, but this would elevate the risk of paraplegia by covering additional intercostal vessels. Therefore, the shortest available graft was chosen. The graft was postdilated with the trilobe balloon catheter to ensure expansion of the stent graft expansion was deemed adequate, thus no balloon postdilation was done to minimize the risk of aortic rupture. The proximal neck was 2-cm long and the left subclavian artery was not covered. The final diameter of the stented aorta at the coarctation site was increased to 18 mm, and there was only a pressure gradient of 10 mm Hg. The patient was discharged after 2 days on diuretics and enalapril for his congestive heart failure. Follow-up computed tomography angiogram 3 months later revealed no endoleak and stable aortic dimensions and graft position. After 3 weeks, the patient underwent successful double-valve surgery, with triangular resection of the P2 segment of the posterior leaflet mitral valve and 32-mm semiring annuloplasty (Cosgrove Edwards (Edwards Lifesciences, Irvine, CA), and replacement of the aortic valve with a 25-mm On-X mechanical prosthesis (Cryolife, Kennesaw, GA). He was discharged on the fifth postoperative day with warfarin anticoagulation. Today, 9 months after his surgery, he is asymptomatic and free of postoperative complications with an ejection fraction of 40%.

## Discussion

CoA typically manifests in infancy with congestive heart failure. Approximately, 15 to 20% of patients remain asymptomatic until adulthood when CoA leads to secondary arterial hypertension. In this case, the diagnosis was missed until later in life when the patient presented with congestive heart failure. In addition to the coarctation, the patient was found to have severe mitral valve regurgitation and severe aortic insufficiency due to bicuspid aortic valve. To minimize the risk of operative repair, the decision was made for staged endovascular CoA repair followed by aortic valve replacement and mitral valve repair.

To the best of our knowledge, this is the first report of TEVAR for CoA followed by double-valve surgery in an adult with cardiac decompensation. Many approaches have been used to treat CoA in adults including open surgical repair, balloon angioplasty, and intravascular self-expanding or balloon-expandable bare-metal stents (BMS). 7

Several reports have shown that TEVAR is a safe and effective approach to treat primary and recurrent CoA, as well as complications of prior CoA repair. 4 5 6 According to multicenter registry analyses, endovascular treatment is performed using balloon-expandable covered stents in approximately 50% of adult patients with CoA, stent grafts in 40%, and balloon-expandable uncovered stents in 10% of this particular patient population. 8

Advantages of balloon-expandable covered stents include wider commercially available TEVAR devices in various sizes and lengths that can accommodate different proximal and distal choice of size, availability of tapered TEVAR grafts to accommodate proximal–distal aortic diameters and lengths, increased conformability in steep or angulated aortic arch anatomy, and ability to exclude associated pseudoaneurysm or aneurysm. 4 5 6 Potential challenges with TEVAR for CoA include inadequate radial force, unnecessary length of aortic coverage, and the potential difficulties to retrieve the dilator tip.

Complications occur in approximately 10% of adult patients undergoing TEVAR for CoA. These include aortic dissection, intraoperative rupture, endoleak, peripheral embolic episodes, and access site hemorrhage among others. In large series, freedom from reintervention and observed survival rates at 5 years are 85 and 90%, respectively. 8

In light of these data, we opted to repair our patient's aortic coarctation with TEVAR. A few weeks later, successful double-valve surgery was performed. This staged approach led to excellent postoperative results and the patient remained free of symptoms at the time of latest follow-up. This unique case provides evidence supporting TEVAR as a viable method to preoperatively stabilize adults presenting with heart failure in the setting of CoA and complex valvular disease.
